# Challenges in the Management of HIV-Infected Malnourished Children in Sub-Saharan Africa

**DOI:** 10.1155/2012/790786

**Published:** 2012-03-01

**Authors:** Indi Trehan, Bernadette A. O'Hare, Ajib Phiri, Geert Tom Heikens

**Affiliations:** ^1^Department of Paediatrics & Child Health, University of Malawi College of Medicine, Private Bag 360, Chichiri 3, Blantyre, Malawi; ^2^Department of Pediatrics, Washington University in St. Louis, Campus Box 8116, One Children's Place, St. Louis, MO 63110, USA

## Abstract

Infection with HIV, and oftentimes coinfection with TB, complicates the care of severely malnourished children in sub-Saharan Africa. These superimposed infections challenge clinicians faced with a population of malnourished children for whose care evidence-based guidelines have not kept up. Even as the care of HIV-uninfected malnourished children has improved dramatically with the advent of community-based care and even as there are hopeful signs that the HIV epidemic may be stabilizing or ameliorating, significant gaps remain in the care of malnourished children with HIV. Here we summarize what is currently known, what remains unknown, and what remains challenging about how to treat severely malnourished children with HIV and TB.

## 1. Background

An estimated 19 million children are severely wasted in developing countries—malnutrition is responsible for 11% of the total global disease burden and 35% of child deaths worldwide [1]. In some regions, notably sub-Saharan Africa, human immunodeficiency virus (HIV) infection poses an added challenge to the care of malnourished children. While the clinical context and interventions for many common causes of childhood mortality worldwide have been addressed over the last decade [2], the management of severe wasting disease and malnutrition in children—particularly in those infected with HIV and/or tuberculosis (TB)—remains poorly addressed [3]. This population of HIV- and TB-infected malnourished children is in many ways very different from the uninfected population for which international malnutrition guidelines [4–6] were originally developed.

In sub-Saharan Africa, the epidemiology of severe malnutrition has shifted to one where an increasing percentage of children requiring hospitalization is composed of those who are HIV infected or HIV exposed—often coinfected with TB—with case-fatality rates still as high as 20–50% [7]. Meanwhile, ready-to-use therapeutic foods (RUTFs) that facilitate effective home-based therapy have resulted in recovery rates for uncomplicated severe malnutrition approaching 90% [8–10], although the recovery rates remain much lower for those children with HIV [11].

In this paper, we present some of the challenges and unanswered questions in the management of malnourished children with HIV (and often TB) and summarize our approach to managing these problems in the absence of clear data to guide us.

## 2. The Magnitude of the Problem

 The average estimated HIV prevalence in 2009 for African adults between the ages of 15 and 49 is about 4.7% [12], with a range from 0.1% to 26% depending on the individual country (Table 1). Meanwhile, there are 137 million children under the age of 5 in sub-Saharan Africa, of whom 12.3 million are wasted. Meanwhile, some 2.3 million children aged 0–14 in the region have HIV [13], and undoubtedly there is significant overlap in these two populations. An estimated 5% of the region's under-five mortality is due to HIV [14], but this is as high as 35% in South Africa [12].

**Figure 1:**
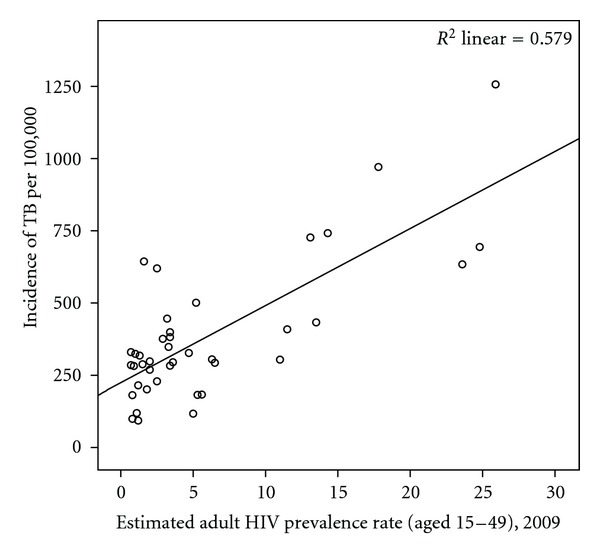
The association between HIV prevalence and TB in sub-Saharan Africa.

Assessing the magnitude of TB incidence in children is less straightforward. The incidence of TB in the under-five age group can be estimated to be half of the adult male incidence and similar to the adult female incidence; following this, then there are at least 330,000 cases of TB each year in children under 5 in sub-Saharan Africa [16]. However, the incidence of TB among HIV-positive children is almost 1600 per 100,000, with a 24-fold higher risk of developing culture-confirmed TB in HIV-positive infants compared to HIV-negative infants [17]. Taking this into account would bring the total number of cases closer to 350,000 annually. Some 30% of all culture-confirmed TB cases who have had an HIV test are found to be HIV positive [17]. Given this and the fact that adults with HIV have an approximately 10% risk per year of developing TB, it is not surprising that countries with high HIV prevalence also have higher prevalence of TB [18] (Figure 1).

Children in sub-Saharan Africa who are HIV infected or HIV exposed are significantly more likely to be stunted, wasted, and underweight [19]. The high prevalence of HIV and TB in sub-Saharan Africa has created a large population of children who are malnourished and infected with both HIV and TB. In the long run, the most effective and efficient method of decreasing the population of HIV-infected children with malnutrition is an aggressive approach to the prevention of mother-to-child transmission (PMTCT) [20], and it is encouraging that a new emphasis has been placed on eliminating new cases of HIV in children [21]. A variety of implementation strategies to achieve this goal are available, and there is opportunity for innovative approaches to be developed locally. For example, Malawi has recently embarked on an aggressive plan to treat all HIV-positive pregnant and breastfeeding women with ART for life (referred to as Option “B+”) [22]. The wide-scale rollout of ART in Malawi in general has also been linked to decreasing rates of TB [23], and we can anticipate that this benefit will continue as more active approaches to HIV control continue to expand, including more regular systematic tracking of patients not yet on ART. Despite these gains, we will continue to be faced with large numbers of malnourished children with HIV—and often TB also—unless and until that laudable goal is reached.

## 3. The Altered Clinical Presentation of Malnutrition in Children Infected with HIV

 There are a number of clinical features that may be helpful in identifying those malnourished children who are also infected with HIV [24]. While HIV-infected children can present with either kwashiorkor (edematous malnutrition) or marasmus (severe wasting), just as those without HIV, a disproportionately larger number of children with HIV present with marasmus. Relative to HIV-uninfected children, malnourished children with HIV tend to be even more stunted and underweight. They more often present with severe oral and esophageal candidiasis, complicating attempts at therapeutic feeding. Greater susceptibility to a variety of infections, for example, cutaneous infections from the skin breakdown often seen with kwashiorkor, means they will often have protracted clinical courses and require more aggressive antimicrobial therapy, wound care, and have a higher caloric requirements. One of the most challenging and frustrating complications faced is persistent HIV-associated diarrhea, for which no specific therapy has been developed and for which no clinical trial evidence yet exists to guide optimal management. A diagnosis of HIV should be strongly suspected in unusually young malnourished children (e.g., those under 6 months of age), in unusually old children (e.g., those over 5 years of age), and in those who do not respond appropriately to nutritional interventions, in addition to the usual suspicion that arises if a child presents with an opportunistic infection such as TB or *Pneumocystis jirovecii* pneumonia (PCP).

Response to therapy is also less predictable and less well-understood in HIV-infected children. Decreased food intake leads to wasting, with an associated reduction in organ system function and an increase in susceptibility to environmental perturbations and stress [25]. Since most of the metabolic responses described in severe malnutrition are based on children without HIV, the responses in HIV-*infected* malnourished children remain largely unknown [26]. When TB or other infections are further superimposed, even less is predictable [27].

## 4. Challenges in Community-Based Care for Severely Malnourished Children with HIV

 The vast majority of children with severe malnutrition can and should be treated as outpatients [8, 9]. The advent and widespread acceptance of RUTF has revolutionized the care of severely malnourished children over the last decade, making it possible to treat children in the community setting. This has relieved much of the burden on inpatient nutritional rehabilitation units (NRUs), whose care can then be reserved for children with complications such as superimposed infections or protracted diarrhea requiring intensive rehydration. Community-based management is now considered the standard of care for children with uncomplicated malnutrition—which accounts for more than 90% of cases of severe malnutrition—who demonstrate an appropriate appetite and have reliable caregivers [10].

 In our practice, we have observed that a large percentage of HIV-positive children have an episode of severe malnutrition as their first AIDS-defining illness. Given that children with HIV and severe malnutrition invariably have lower nutritional recovery and higher mortality rates than their HIV-negative counterparts [28] and that those who do recover take longer to achieve nutritional recovery [10], it is imperative that voluntary HIV testing and counseling be offered to all children with severe malnutrition in order to identify those with HIV. Children diagnosed with HIV should then be referred for PCP prophylaxis and ART as soon as possible—there is no evidence to indicate that delaying ART is of benefit to this population of children, with regards to either decreasing rates of the immune reconstitution inflammatory syndrome (IRIS) or to the adverse metabolic effects of ART. In fact, decreasing the metabolic and energy demands placed on the child's physiology due to uncontrolled HIV will in general speed up nutritional recovery as well [29].

 In practice, there is often a delay in initiating ART in the community setting, due to delays in testing, counseling, drug procurement, and other steps in the process. Ideally, malnutrition and HIV services should always be available, complementary, and well coordinated in any normal daily clinic—rather than caretakers being required to return on multiple separate occasions to have each illness addressed individually. While this may initially be challenging in large health centers, the strong link between malnutrition and HIV makes this a worthy goal to work towards in our opinion. Linking these services together, providing efficient clinics, and decreasing the number of follow-up visits necessary for routine care are all efforts that may help increase retention and decrease the number of children lost to followup.

## 5. Challenges in the Hospital Care of Severely Malnourished Children with HIV

 In the past, NRUs typically admitted sick severely malnourished children mostly during periods of food insecurity or in the postweaning period [30]. In sub-Saharan Africa, we now admit many HIV-infected malnourished children outside of these traditionally high-risk periods. These children frequently present with many superimposed infections, including (persistent) diarrhea, pneumonia, PCP, TB [31], extensive cutaneous infections, and oral and gastrointestinal candidiasis [32]. Case fatality rates are high in these children, especially those with profuse diarrhea, and their response to standard management protocols is poor [33]. Extremely wasted and stunted adolescents, previously seen only rarely outside the setting of famine, are now admitted frequently for nutritional recovery and often present with chronic HIV-related pathology such as chronic lung disease [34]. The percentage of children who are readmitted has also increased from 1-2% to more than 10% [35].

 In Zambia and Malawi, for example, more than half of patients admitted to many NRUs these days are HIV positive, with case fatality rates of 40% or higher [31, 36]. Mortality in severe malnutrition is already known to be elevated whenever a child presents with superimposed infections [37, 38] and metabolic maladaptation.

 Since early in-hospital mortality is high [35], improvements in initial treatment strategies depend on improved knowledge of the most common causes of infection and antibiotic sensitivities [39], pharmacokinetics of anti-infective medications in malnourished children, and potentially complex drug interactions and toxicities (e.g., ART and anti-TB therapy [40]). Bacterial susceptibility to first-line antibiotic treatment varies between centers, and the choice of empiric antimicrobials needs to be modified to suit local resistance patterns [39]. The effect of widespread usage of co-trimoxazole for PCP prophylaxis is already leading to resistance among the most common pathogens [41, 42]. In our setting, we see nearly 100% resistance to co-trimoxazole by *Streptococcus pneumoniae* [43], leading to increased use of second-line agents.

The metabolic and nutritional needs of HIV-infected children are not well known [44, 45]. In HIV-uninfected malnourished children, appetite is useful to guide nutritional rehabilitation, but this seems not to be the case in HIV-infected children since persistent anorexia is common. World Health Organization (WHO) guidelines for the management of children hospitalized with severe malnutrition [4, 5] provide little specific guidance for the treatment of malnourished children with HIV [46]. Evidence from clinical trials on how the management of severe malnutrition should be modified for children with HIV is lacking, and treatment protocols remain based almost entirely on expert opinion and extrapolation from other populations. Given this absence of evidence, it is our practice to initially stabilize children with milk-based formulas such as F-75 [4]. These children are generally ill enough to require urgent stabilization of other physiological parameters as well, including correction of hypoglycemia, hypothermia, dehydration, and electrolyte imbalances. The use of empiric antibiotics and antihelminthic medication for presumed active infections and a presumed immune compromised state is often advocated as well [5]. After this initial stabilization phase, feedings can be advanced to a regimen of F-100 or RUTF, the latter being preferred so that children can be discharged to complete their care at home (thereby reducing crowding in the ward and the risk of nosocomial infections) that much sooner [9]. Unlike those without HIV, children with HIV frequently have difficulty tolerating an advancement of their feedings, and case fatality rates remain high during this period [47]. Evidence on the optimal feeding regimen to be used for HIV-infected children is lacking, and the choice is thus generally based on local preference and resources, with consideration given to the rate of weight gain and to adverse events such as persistent hypoglycemia and an increase in osmotic diarrhea or clinical signs of heart failure.

 Suitable feeding and rehydration regimens are still needed for the severe diarrhea commonly observed prior to and during rehabilitation of HIV-infected severely malnourished children, which is often associated with hypoglycemia [48, 49] and high case fatality rates [33, 50]. In the past, adequate treatment regimens were developed for HIV-uninfected children to reduce diarrheal morbidity and mortality, induce catch-up growth, and improve nutritional outcomes [51]. Modified or improved rehydration regimens for HIV-infected children in this context may be helpful, although no specific regimens are available at this time.

The spectrum of organisms associated with bacteremia in this population [39, 52, 53] supports the importance of mucosal translocation [54] as the inciting event. Marasmic children in one Zambian study showed lower CD4 counts compared to children with edematous malnutrition (kwashiorkor), correlating with the protracted nature of diarrhea observed in these children from opportunistic enteropathogens such as* Cryptosporidium* [27]. Severe wasting makes the clinical assessment of dehydration difficult, so the presence of metabolic acidosis and lethargy are often the clinical indications available to prompt resuscitation. Unfortunately, there are also currently inadequate data on the optimum regimen of supportive care (e.g., for shock) in the malnourished child who has adapted to a reduced body mass and organ system function [38, 55].

 There remains variation in how severely malnourished infants under 6 months of age are treated—explicit recognition of this population is only slowly emerging, and their mortality remains significantly higher than children older than 6 months [1]. Appropriate dietary therapies are needed for this increasing population, as the standard F-75 and F-100 formulas are likely unsuitable [6]. The poor socioeconomic background of these children often makes the use of commercial formulas for continued care as outpatients impractical [56].

## 6. Challenges with Initiating ART in Children with Severe Malnutrition

 The optimal timing, regimen, and dosing of ART in the inpatient population of children with complicated malnutrition remain guided primarily by expert opinion due to a lack of prospective trial evidence [6, 29]. A number of observational studies have shown that HIV-infected children started on ART with more severe wasting have higher rates of mortality than those with less wasting [57–59], but no trial evidence exists to suggest that waiting until a child's nutritional status improves correlates with improved outcomes. In fact, an important recent retrospective study suggests that malnourished children who start ART promptly have higher rates of nutritional recovery and weight gain than those in whom ART is delayed [60].

A study in Zambia has shown that simply improving the nutritional status of severely malnourished HIV-infected children is insufficient to improve their immunological status without ART [27]. In fact, excellent responses in CD4 count and viral load have been demonstrated among those with severe malnutrition who do receive ART, just as in those with better baseline nutritional status [58]. In a cohort of Zambian children where 59% of the children were initially underweight and 72% stunted when starting ART, lasting improvements in both weight and height were observed, with weight-for-age Z-scores increasing during the first 6 months of treatment before stabilizing and with height-for-age Z-scores increasing consistently over time. In this cohort, children who were the most underweight experienced the greatest increases in weight [61], likely a regression to the mean phenomenon. This initial correction of wasting followed by a lasting correction of stunting has been documented in the rehabilitative process of HIV-uninfected malnourished children previously [51, 62]. In sum, it is clear that severely malnourished children are indeed able to respond appropriately to ART and nutritional supplementation in terms of both nutritional and immunological recovery. Therefore, these lifesaving medications should not be delayed, and child health systems should embrace this in a programmatic manner.

 Admittedly, optimal timing for starting ART remains controversial due to concerns over IRIS [36, 63]. One case-control study comparing baseline factors related to the development of IRIS did show that children with at least one form of malnutrition (among stunting, wasting, and underweight) were more likely to develop IRIS [64]. Nevertheless, there were no fatalities among those that developed IRIS, and none required treatment interruptions from their ART. At the same time, delays in initiating ART while children are treated for TB has been shown to be detrimental to overall clinical response and mortality, particularly in children with severe immune suppression [65]. Although the clinical presentation is not typical of any known ART toxicity, it is possible that ART initiated in children with severe malnutrition and immunosuppression may lead to a clinical deterioration and a syndrome mimicking kwashiorkor [63]. The low levels of circulating antioxidants and high levels of lipid peroxidation products seen in untreated TB patients [66] may be components of the perturbed physiologic state malnourished children find themselves in. These may further be part of their reductive adapted state, leading to a kwashiorkor-like presentation of IRIS. In the end, the data on this are relatively minimal and inconclusive, and ultimately clinical judgment will have to be applied on a case-by-case basis [67].

 The alterations in body composition (decreased fat and lean body mass) and metabolic functions (changes in renal, hepatic, mitochondrial toxicity, and antioxidant capacity among others) in children with severe malnutrition has led to the concern that standard dosing of ART in these children may be inappropriate. On the one hand, these metabolic alterations perhaps lead to subtherapeutic drug levels that may contribute to viral resistance, while on the other hand these medications may be given at levels too toxic for these fragile children to tolerate safely. Unfortunately, only limited data exists to guide dosing recommendations for ART in malnourished children. One observational study on the pharmacokinetics of nevirapine in 37 Malawian children showed that suboptimal dosing was not more prevalent among moderately malnourished children [68]. As far as we are aware, there are no studies that present data on toxicity or other adverse effects from ART in severely malnourished children nor is there any literature to guide which initial ART regimen is best to start.

Further research evaluating the effects of ART in children with severe malnutrition is clearly necessary, and initial studies are underway in Malawi. High-priority areas for study include the optimal choice and timing of the initial ART regimen, an assessment of toxicity and pharmacokinetics, and an evaluation of the incidence and risk factors for IRIS.

Implementation challenges in starting and maintaining children on ART also persist and are found throughout the chain of care in sub-Saharan Africa, especially in rural areas. We have firsthand observed inconsistent supplies of testing kits, reagents, and trained staff to provide voluntary testing and counseling. Once testing is done and HIV-infected children identified, there are often further delays in referrals for group counseling, individual counseling, and ultimately initiation of therapy—all of which require trained staff and clinic facilities—and which often occur in different locations and at different times, placing a further burden on caretakers. Finally, supplies of ART and follow-up clinical and laboratory monitoring remain inconsistent, leaving patients vulnerable to treatment interruptions, which could compound HIV control efforts by increasing rates of viral drug resistance and spread. Aggressive investments in expanding national HIV prevention and treatment programs [69] must also address these and other challenges, which are often faced most severely by rural health centers.

## 7. Challenges in Detecting Tuberculosis in Malnourished Children with HIV

 TB is notoriously difficult to diagnose in children. When a child has both severe malnutrition and HIV, diagnostic testing becomes even more difficult and, ultimately, also that much more important [70]. The tuberculin skin test (TST), long the first-line screening test, suffers from poor sensitivity and specificity. Sensitivity suffers further when the child is immunocompromised from HIV and severe malnutrition, which blunt the type IV hypersensitivity reaction needed to demonstrate a reactive TST. Given the high background rate of TB in sub-Saharan Africa (with childhood HIV-TB coinfection rates as high as 50% [71]), diagnosis is often left to clinical judgment in those children with an exposure history or with signs or symptoms or perhaps a chest radiograph suggestive of TB [72]. Undoubtedly, this leaves many children with TB undiagnosed and untreated, as well as many children without TB treated unnecessarily. Local health systems are unfortunately often too overburdened to pursue contact tracing when a case of TB is identified, but this remains an important component of the public health approach to minimizing the spread of TB by providing isoniazid preventive therapy (IPT) to children exposed to adults and older siblings with active TB. IPT can also be considered for all HIV-infected children and adults not yet on ART, as has recently been recommended in Malawi [69].

 Aside from traditional means of improving the microbiological sensitivity of TB diagnosis in children such as the string test or induced sputum [71], newer nucleic acid amplification methods such as the Xpert MTB/RIF test show great promise as screening tools for TB, even in malnourished children from a population with a high rate of HIV [73]. The relative affordability of these tests, along with the speed with which results are available, has the potential to revolutionize TB care and has recently led to their endorsement by the WHO [74]. Nevertheless, implementation of such testing is likely to remain years away in most of sub-Saharan Africa, and thus clinicians will need to continue to have a low threshold for diagnosis of TB based on clinical and epidemiological suspicion. As with the challenges involving ART described earlier, no evidence exists on whether the dosing, timing, or medication choice of anti-TB regimens need modification in this population of children.

## 8. Looking Ahead: An Agenda for Research

 Undernutrition, HIV, and TB all contribute to a large proportion of child deaths worldwide, and this high mortality rate is as avoidable as it is complex. As progress in the rollout of ART and PMTCT accelerates in sub-Saharan Africa, there are hopeful early signs that the number of children with severe malnutrition and HIV will decrease in the future. Nevertheless, for the foreseeable time, this population of patients will continue to challenge practitioners on the frontline, and the evidence base for practice is limited.

A number of lingering questions remain that deserve the attention of researchers in this setting (Table 2). Aside from pursuing these specific questions in the context of patient-oriented clinical trials, implementation of any findings remains a further challenge for overburdened local health systems. Operational research on the best means of implementing these findings and how efficacious these findings would be in practice would thus also be useful in order to inform the most cost-effective therapy for HIV-infected children with malnutrition.

## Figures and Tables

**Table 1:** HIV prevalence in adults 15–49 years old in Africa [12].

Prevalence of HIV	Number of countries	Country
0–0.9%	7	Algeria, Comoros, Eritrea, Madagascar, Mauritania, Niger, Senegal
1–4.9%	19	Angola, Benin, Burkina Faso, Burundi, Central African Republic, Chad, Congo, Côte d'Ivoire, Gambia, Ghana, Guinea, Guinea-Bissau, Liberia, Mali, Mauritius, Nigeria, Rwanda, Sierra Leone, Togo
5–9.9%	6	Cameroon, Equatorial Guinea, Gabon, Tanzania, Kenya, Uganda
10–19.9%	6	Malawi*, Mozambique*, Namibia*, South Africa*, Zambia*, Zimbabwe*
20–30%	3	Botswana*, Lesotho*, Swaziland*
Missing	5	Cape Verde, Democratic Republic of Congo, Ethiopia, Sao Tome and Principe, Seychelles

* These 9 countries account for 50% of the global burden of HIV-associated TB [15].

**Table 2:** Selected examples of open research questions in the care of HIV-infected children with severe malnutrition.

ART in malnourished children Appropriate medication regimen? Changes based on maternal nevirapine exposure? Appropriate dosing? Optimal timing of initiating ART? Adverse effects of ART in malnourished children? Clinical or laboratory markers of adverse effects? Risk factors or preventive measures for IRIS? Interactions with TB medications?	

Diarrhea in HIV-infected malnourished children Optimal rehydration regimen? Modified rehydration solutions (both oral and parenteral)? Role for antibiotics or antiparasitics? Role for zinc, glutamine, or probiotics?	

Nutritional therapy in HIV-infected malnourished children Need to modify screening criteria for enrollment into nutritional care? Need to modify criteria for younger (<6 mos) and older (>5 yrs) children? Optimal initial choice of therapeutic food? Optimal choice of therapeutic food during transition phase? Need to modify criteria for graduation from nutritional care?	

Adjuncts to nutritional therapy in HIV-infected malnourished children Vitamin A? Empiric choice of antibiotics and antiparasitics?	

TB diagnosis in HIV-infected malnourished children Accuracy of traditional methods for diagnosis (e.g., chest X-ray, TST)? Accuracy of newer methods for diagnosis (e.g., Xpert MTB/RIF)?	

Operational challenges How to implement the above findings and guidelines at each level of care? Prenatal and perinatal care? Rural health center? District hospital? Central referral hospital?	
